# Readability of Information Related to the Parenting of a Child With a Cleft

**DOI:** 10.2196/ijmr.4210

**Published:** 2015-07-08

**Authors:** Nanci De Felippe, Farnaz Kar

**Affiliations:** ^1^ School of Dentistry Division of Orthodontics University of Minnesota Minneapolis, MN United States

**Keywords:** cleft lip, cleft palate, parenting, readability, literacy

## Abstract

**Background:**

Many parents look to various sources for information about parenting when their child has a cleft lip and/or palate. More than 8 million Americans perform health-related searches every day on the World Wide Web. Furthermore, a significant number of them report feeling “overwhelmed” by the language and content of the information.

**Objective:**

The purpose of this study is to determine the readability of information related to parenting a child with cleft lip and/or palate. It was hypothesized that the readability of such materials would be at a level higher than 6th grade.

**Methods:**

In February of 2012, a Web-based search was conducted using the search engine Google for the terms “parenting cleft lip and palate.”

**Results:**

A total of 15 websites, 7 books, and 8 booklets/factsheets (N=30) entered the readability analysis. Flesch-Kincaid Grade Level, Fog Scale Level, and Simple Measure of Gobbledygook (SMOG) index scores were calculated. The reading level of the websites and books ranged from 8th to 9th and 9th to10th grade, respectively. The average reading level of the booklets/factsheets was 10th grade. Overall, the mean readability of the media resources analyzed was considered “hard to read.” No statistically significant mean difference was found for the readability level across websites, books, and booklets/factsheets (Kruskal-Wallis test, significance level .05).

**Conclusions:**

When considering websites, books, booklets, and factsheets analyzed, the average readability level was between 8th and 10th grade. With the US national reading level average at 8th grade and the general recommendation that health-related information be written at a 6th grade level, many parents may find the text they are reading too difficult to comprehend. Therefore, many families might be missing out on the opportunity to learn parenting practices that foster optimal psychosocial development of their children.

## Introduction

The birth of a child can have great impact in any family system, let alone when it is the birth of a child with a disability or a facial difference such as a cleft lip and/or palate (CLP). In this situation, parents may not only have to adjust to the expected demands of parenthood but also manage challenges resulting from their child’s congenital anomaly [1]. Parents anticipate and worry about countless stressors, including multiple reconstructive surgeries, feeding hurdles, dental agenesis and malalignment, facial-skeletal disharmony, speech impairment, strained social relationships, and compromised self-image for the child [2,3].

In addition to concerns regarding the physical complications, parents are often overwhelmed by the task of seeking information to better understand their child’s condition, treatment, and management. One resource that has the potential for providing an abundance of information is the Internet, which is being increasingly used to answer questions and gain knowledge. A 2006 study by Fox [4] showed that over 100 million Americans used the Internet for health information searches in that year, with 8 million Americans searching the Web each day. She also observed that 514 individuals (25% of the sample investigated) reported feeling “overwhelmed” when acquiring online information on health-related topics [4]. This overwhelming sensation could be due to the vast number of resources, to problems with understanding the content of the website, and also its trustworthiness.

Comprehension is an important factor and one that is often overlooked as families are encouraged by health care professionals and those who provide other services to the family to turn to resources such as the Internet for support and information. Potentially useful information that could educate and improve parenting skills is often serving no practical purpose due to its readability level. In 2001, Berland [5] wrote “One must be able to comprehend the material in order to be able to utilize it.” The utilization of the information to better care for a child with a cleft is ultimately the main goal.

According to Graber et al [6], the reading level of a person in the general population is usually lower than that of the final grade level he/she completed. Furthermore, those who navigate the Web in search of health-related information face another layer of difficulty: clinical terminology (ie, medical and dental). According to D’Alessandro et al [7], the US national reading level is in the range of 8th to 9th grade. They recommended that health-related websites and printed literature should aim for a 6th grade reading level. This recommendation has been adopted by the Center for Disease Control and Prevention (CDC), the American Medical Association (AMA), and the National Institutes of Health (NIH). Finally, they also concluded that even those individuals with higher readability levels prefer to read easier documents [7].

Parenting includes, but is not limited to, everything that supports the physiological and social development of a child besides the basic responsibility of providing shelter and food. Literature on parenting is vital to those wishing to have guidance with parenting practices or looking for answers on how to deal with health-related, social, or psychological issues when raising a child. Parenting practices are all the actions parents take to socialize children’s behaviors and, as such, they primarily influence the shaping of children’s behavioral adjustment. Resources that teach and enhance parenting practices allow parents to promote ideal development of their child by optimizing their potential [8]. Parents also have the power to create an environment that rears a child in a desired direction [9]. A study conducted by Klein et al [10] showed that mothers, specifically of children with craniofacial anomalies, experienced higher levels of emotional and social adjustment in comparison to parents of unaffected children and, therefore, demonstrated greater need to have their parenting practices coached. Since CLP children are at higher risk to develop psychosocial adjustment problems, it is possible that their parents are using the Internet as a resource to avoid, combat, or decrease the frequency of such challenges [9]. As such, accessible and understandable literature on parenting can provide some stress relief and much needed guidance.

The purpose of this study is to determine the readability of information related to parenting a child with CLP available to the public via the Internet. Our hypothesis is that the readability of such materials is greater than the 6th grade level recommended by the CDC, AMA, and NIH.

## Methods

This study reports the findings of a Web search conducted using the Google search engine in February, 2012 using the terms “parenting cleft lip and palate.” A total of 1,980,000 links showed up in 0.39 seconds. The first 5 pages of results were analyzed based on the findings described by Jansen and Spink [11] who observed that most users explore the results displayed in the first page only. We expanded our analysis to include the first 5 pages to account for computer and display variances in font size and formatting. The first 5 pages of our Google search yielded a total of 74 links. The same search was conducted a few years later (March 25, 2015 yielded 176,000 results in 0.43 seconds and April 24, 2015 yielded 120,000 results in 0.45 seconds) and a different pattern of information was found on the first 5 pages because the Google algorithms, programs, and formulas for analyzing individual Web pages had changed over the years. The most remarkable changes observed in the 2015 searches were (1) the top 3 websites were sponsored links, as opposed to 2012 where all sponsored links remained on the right side of the organic results, (2) more books (3x) were present, (3) more blogs (2x) were present, (4) more research papers (1.5x) were present, and (5) there was fewer irrelevant information, which lead to a 27% increase (94/74) in usable resources.

Relevant links were the ones that included information about orofacial clefts, craniofacial anomalies, and/or facial differences in general. Irrelevant links included repetitions, advertisements, and resources not related to either craniofacial anomalies or facial difference. After the exclusion of irrelevant information, 42 links were analyzed (Multimedia Appendix 1). Of those, 38% (16/42) included information on parenting a child with CLP. Of those, 1 was protected against copying and pasting and, therefore, a total of 15 (36%, 15/42) websites entered the readability evaluation.

**Figure 1:**
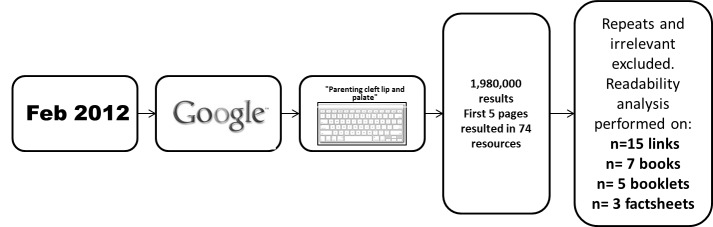
Diagram of data collection.

The links that offered written resources such as books, booklets, and factsheets were also recorded. This Google search led to the finding of 25 books and 18 booklets/factsheets. Of those, only the books (32%, 8/25) and booklets/fact sheets (44%, 8/18) addressing the “parenting” theme entered the readability analysis (Multimedia Appendices 2 and 3). After directly contacting the author of one of the books, it was learned that its reprints were no longer being published and we were thus unable to perform the readability test on it. Therefore, a total of 7 books were included. A thematic analysis of the content of each resource was performed so that patterns of information could be recorded. After familiarization with the data, initial codes were generated and generic themes emerged from the preliminary analysis. Lastly, a list with the most frequent themes (ie, author, country of origin, information specific on cleft, terms and definitions, etiology, team approach, feeding, surgery, orthodontics, speech, hearing, links and paths to request information, social support, as well as information on parenting practices) was created. Websites, books and booklets/factsheets had their content analyzed for the presence or absence of each theme. The data collection process is shown in Figure 1.

Readability for the 15 websites, 7 books for parents, and 8 booklets/factsheets was tested using the Flesch- Kincaid Grade Level, the Fog Scale Level, and the Simple Measure of Gobbledygook (SMOG) index. These tests were selected to be used in this study for the following reasons (1) they were readily accessible on the Internet and free of charge, (2) they have been used in sociology, healthcare, and publishing/media literature [6,7,12-16], (3) they were fairly easy to use, and (4) their formulas complement each other (ie, the general recommendation is to use them together to improve validity of the results) [12,13]. While some readability formulas are validated against various tests of comprehension, the most common being McCall-Crabbs criterion [17], there is no gold standard readability test. For instance, the Flesch-Kincaid readability formula calculates the average number of words per sentence and syllables per word, then inputs those numbers into the Flesch-Kincaid Readability Age (FKRA) formula:

FKRA=(0.39 × average sentence length) + (11.8 × average number of syllables per word) − 15.59

Average sentence length is calculated by dividing the number of words by the number of sentences and average number of syllables per word is calculated by dividing the number of syllables by the number of words [14].

The Fog Scale formula calculates the average sentence length by dividing the total number of words by sentences in a sample portion from the text that has ≥100 words. It then calculates the percentage of “hard” words by dividing the number of words that have ≥3 syllables (and that are not proper nouns or hyphenated words) by the total number of words in the sample portion [16]:

Grade level=0.4(average sentence length + percentage of hard words)

Finally, the SMOG readability formula selects 10 consecutive sentences from the beginning, middle, and end of the text. From these sentences the number of words with ≥3 syllables is counted and the square root of this number is rounded off to the nearest 10 [18]:

SMOG grade=3 + √polysyllable count

In 2010, Burke and Greenburg [19] compared several readability formulas and recommended that, especially for health-related literature where 100% comprehension is a goal, a combination of ≥2 formulas, including the SMOG, should be used.

Website URLs were copied from an Excel spreadsheet into a browser using the latest available version of Microsoft Office Word software. Once the Web page was displayed, the text from that link was copied in its entirety. The text was copied into a text box available on the online readability calculator as previously described by Antonarakis and Kiliaridis [12]. The first and last 50 words of each chapter for all 7 books were typed out into a Microsoft Word document and later pasted into the text box available on the online readability calculator. The first and last 100 words of each booklet and factsheet were typed into a Microsoft Word document and each was analyzed separately using the method mentioned above.

## Results

Only 16 websites (38%, 16/42) mentioned direct or indirect guidance for parenting practices. Of these, 15 (94%, 15/16) entered the readability analysis; the one remaining link was not used because it was protected against copying of information. Overall, the analysis of the Fletch-Kincaid resulted in a mean score of 8.93, which correlated to a 9th grade reading level. The Fog scale gave a mean score of 11.50, which correlated with a "hard to read" readability level. Finally, the SMOG scale resulted in a mean score of 8.24, implying an 8th grade reading level.

Of the books, 17 (69%, 17/25) were written for children and 8 (32%, 8/25) were written for parents. Of the total, only 6 (24%, 6/25) were not specifically written for those with orofacial clefts. All books written for parents included either direct (20%, 5/25) or indirect (12%, 3/25) parenting advice. One book could not be used since it was no longer published. The readability analysis for the books (n=7) resulted in a mean Fletch-Kincaid score of 9.76, which correlated to a 10th grade reading level. The Fog scale gave a mean score of 12.54, which correlated with “hard to read”. Finally, the SMOG scale resulted in a mean score of 8.96, implying a 9th grade reading level.

Factsheets (n=3) and booklets (n=5) were analyzed because they contained parenting advice that was either direct (88%, 7/8) or indirect (12%, 1/8). The readability analysis for the booklets resulted in a mean Fletch-Kincaid score of 10.44, which correlated to a 10th grade reading level. The Fog scale gave a mean score of 14.54, which correlated to "difficult to read". Finally, the SMOG scale resulted in a mean score of 10.10, implying a 10th grade reading level.

Even though the booklets/factsheets had the greatest readability scores among the media resources analyzed, when we tested the mean readability difference among the 3 groups using Kruskal-Wallis, we could not find any statistically significant difference (at a power of 80%). This suggests that all 3 groups of media resources presented similar mean reading scores for the 3 tests (Fletch-Kincaid Grade Level, Fog Scale Level, and SMOG Index) (Table 1) and were all considered “hard to read.”

**Table 1:** Descriptive statistics and Kruskal-Wallis results for readability level.

Media Resource	Fletch-Kinkaid Grade Level, mean (SD)	Fog Scale, mean (SD)	SMOG index, mean (SD)	Kruskal-Wallis test, *P* value^a^
Websites	8.93 (2.27)	11.50 (2.32)	8.24 (1.76)	.20
Books	9.76 (3.42)	12.54 (3.91)	8.96 (2.67)	.09
Booklets/factsheets	10.44 (2.43)	14.54 (3.41)	10.10 (2.01)	.10

^a^Significance level at *P*=.05

## Discussion

### Principal Findings

The readability analysis of websites ranked in the top 5 pages of a Google search, as well as books and booklets/factsheets accessed through those links, was performed based on the models proposed by Antonarakis and Kiliaridis and Fitzsimmons et al [12,15]. Google was the search engine of choice because in 2008, Lewandowski [20] noted that users looking for health-related issues perceive it as the best search engine due to its ability to deliver a high ratio of relevant results and descriptions per search.

The Internet is a popular source of parenting information, as well as any consumer-oriented healthcare information, that is convenient and of relative easy access [15]. In agreement with Antonarakis and Kiliaridis [12], we believe that there is an urgent need to guide practitioners and those involved in CLP care towards the most useful, reliable, readable, and complete websites, so that they can direct patients seeking information to these sites. The World Health Organization (WHO) recognizes the problem related on any health topic [21] and has proposed the creation of and supervision of a ‘‘health’’ domain to impose standards of quality on all disclosed materials.

This study did not investigate the quality and/or utility of the information, rather it focused on the readability aspect of the information available to parents. We observed that, overall, the contents of the websites varied greatly in covered themes as well as in quantity. Of all the websites, 29 were loaded with medical technical information, while 13 were blogs and forums of lay people sharing their life experiences. This is a similar finding to Antonarakis and Kiliaridis [12] who also concluded that the information available to CLP families on the Internet is vast and highly variable. The consequence of such abundant and variable sources of information is yet to be determined. However, all of these resources are only useful if the consumer understands their content [15].

With respect to readability, Antonarakis and Kiliaridis [12] found that website information on orthodontics for the CLP population is on average at the 8th to 9th grade level. Our research, which investigated the topic of “parenting practices,” found the level to be slightly higher at a 9th to 10th grade. For instance, the Cleftline website [22], which is one of the most popular websites, has a reading level of 11th grade. On the other hand, the Specialchildren and Café Mom websites [23,24] had reading levels below 6th grade. Interestingly, Specialchildren is a website dedicated for parenting children with special needs, and was most likely designed with the goal of establishing clear communication with families. Café Mom is also a parenting website designed by a marketing corporation (CMI Marketing, Inc) which probably used effective communication strategies in its design.

Our findings for books, booklets and factsheets had a similar range of 9th to 10th grade. Most books written by parents for parents, such as “Children with Facial Difference: A Parent’s Guide” had high reading levels (11th grade) [25]. However, 2 books written by parents for parents were exceptions: “Don’t Despair Cleft Repair” and “An Unconditional Love” [26,27] had scores at the 6th grade level. Books written by experienced doctors, despite the fact that they are routinely recommended by health care professionals, were considered hard to read by an adult based on the readability scores, as compared to US national literacy averages. Dr Berkowitz’ “The Cleft Palate Story” [28], for instance, had the reading level of at/or above college level, while Dr Moller’s book, “Parent’s Guide to Cleft Lip and Palate” [29], scored at an 11th grade level. Likewise, highly recommended and used booklets from the Cleft Palate Foundation scored high on the readability test. It is not uncommon to have these booklets readily available for families in outstanding cleft/craniofacial centers in the United States. The most difficult one to read according to our study, and perhaps one of the most popular ones, titled “Toddlers and Preschoolers” [30], rated at/or above college level. In general, booklets/factsheets had higher reading scores and were not found to be statistically different than the average reading scores for books or websites. Considering these findings, the reading level difficulty poses a problem for a large percent of the population.

When authorship and reading level were analyzed together, it was observed that resources written by parents, especially by those who write well in English such as Terri Mauro (BA in Literature) from the Specialchildren website [23] and Karen Lipman, author of “Don’t Despair Cleft Repair” [26] presented lower grade reading levels. Likewise, the book “An Unconditional Love” [27] written by the experienced mystery writer, Lorraine Barlett, was found to be at an “easy to read” level.

Basic reading level indicates skills necessary to perform everyday literacy activities, such as reading and comprehending information in simple documents, such as charts and forms. Below basic reading level indicates no more than the most simple and concrete literacy skills, such as locating easily identifiable information, and following written instructions in simple documents [31]. The average reading level for the American population [12,7] is 8th grade. It is important to differentiate between an individual’s academic grade achieved and actual reading skill. Studies have demonstrated that one’s reading level is usually lower than his/her highest accomplished academic grade [16,17]. Therefore, it is possible that most of the websites investigated in this study would not be consistent with the readability level of individuals with a high school diploma, which make up approximately 30% of the population (ie, around 42 million adult internet users in 2006) [4].

Parenting a child with CLP can be challenging because of the increased emotional, physical, and social considerations that exist related to the condition in different stages of the child’s life [10]. It is expected and understandable that parents have a thirst for knowledge about their child’s condition and the psychosocial adjustments needed as he/she grows. Knowledge has the potential to play a profound coping role throughout this entire process. Health care professionals are encouraged to provide parents with accurate written and oral information [18] in order to facilitate the learning and coping process. Although this is helpful, many families still turn to the Internet to address unanswered questions and concerns that arise throughout their child’s treatment process [32]. Based on our findings, they are likely to face the challenge of understanding the material due to the difficult readability levels of the vast majority of media resources. In addition to general readability, adding the dimension of health-related vocabulary that is likely unfamiliar to the parent makes the text more challenging to comprehend [7]. As a result, parents do not acquire the guidance and knowledge they are seeking to incorporate in parenting practices, which could benefit their child’s development.

### Conclusions

Most resources tested presented with average reading scores above the US national’s average literacy scores [31]. There is a vast amount of information available, especially with the growth and convenience of the Internet. However, this material may only be useful if patients are able to comprehend it [15,32]. The suggested reading level of information related to CLP should be at the 6th grade level [7]; endorsed by the CDC, AMA, and NIH. Our study found that only 4 resources (13%, 4/30) in compliance with this recommendation. The books “Don’t Despair Cleft Repair” [26] and “An Unconditional Love” [27], written by parents of children with CLP, and the websites Specialchildren and Café Mom specialized in parenting practices and tips to raise children.

When considering the books, factsheets, booklets, and websites analyzed, the average readability level was between 8th and 11th grade. With the US national average at 8th grade, many parents are probably finding the text they read too difficult to comprehend. In agreement with Antonarakis et al [12], we believe that there is an urgent need to guide practitioners and those involved in CLP care towards the most useful, reliable, readable, and complete websites, so that they can direct patients seeking information to these sites.

### Recommendations

There are multiple ways in which this useful material can become more readable and relevant for parents. Some recommendations are (1) the use of short sentences and avoid passive voice, (2) limit medical jargon, explain the root of medical terminology, and break down long medical words [33], (3) avoid ambiguous words, symbols, and quotation marks [13], (4) select familiar words and use them consistently [7,13], (5) use analogies that are familiar and culturally appropriate for the target audience [13,33], (6) instead of real numbers, when conveying statistics use words a such as ‘‘half,’’ or ‘‘one third’’ [13], (7) plan and test websites as well as booklets before releasing/publishing them, (8) use free readability tests available on the Internet to improve the readability level of a text from "hard to read" to the 6th grade level [13,5], (9) use illustrations, pictures, and/or simple drawings as an effective alternative to substitute complex words or terms [5,34], and (10) explain procedures, symptoms, and treatment modalities using plain language in conversation style (eg, making use of a plain language website [35]). Comprehensible material is a necessity to foster confidence and understanding of the anomaly while promoting effective parenting practices in families with children with CLP. It is imperative that organizations test the readability of the content in their websites prior to making them available to the general population.

Illustrations or pictures may also be useful in explaining a technique or self-care procedure to a patient. Key messages can be communicated in a manner that is not demeaning to individuals with low health literacy [31]. As providers develop consumer health materials, readability-assessment tools such as Gunning FOG, SMOG, or Flesch-Kincaid may assist them to edit the writing down to the appropriate reading level. This step provides a quality check to ensure that patient-education materials meet the United States Department of Health and Human Services (USDHHS) reading-level recommendation.

## Appendix

Multimedia Appendix 1 Content analysis of websites. Only the websites containing parenting information are shown.

Multimedia Appendix 2 Content analysis of books. Only the books containing parenting information are shown.

Multimedia Appendix 3 Content analysis of booklets and factsheets. Only the resources containing parenting information are shown.

## Abbreviations

AMA: American Medical Association
CDC: Centers for Disease Control and Prevention
CLP: cleft lip and/or palette
FKRA: Flesch-Kincaid Readability Age
NIH: National Institutes of Health
WHO: World Health Organization
