# Supplementary material for: Readability of Information Related to the Parenting of a Child With a Cleft
Source: Interact J Med Res. 2015 Jul 8;4(3):e14. doi: 10.2196/ijmr.4210 (PMC4526983; doi:10.2196/ijmr.4210)
Supplement: Multimedia Appendix 3 [file ijmr_v4i3e14_app3.pdf]

Booklets and factsheets	Author	Country of origin	Cleft specific	Terms and definitions	Etiology	Team approach	Feeding	Surgery	Orthodontics	Speech	Hearing	Links and request information	Social support
Your baby's first year	Cleft Palate Foundation	USA	+	+	+	+	+	+	+	+	+	-	+
Toddlers and preschoolers	Cleft Palate Foundation	USA	+	-	-	+	-	+	+	+	+	-	+
The school aged child	Cleft Palate Foundation	USA	+	-	-	+	-	+	+	+	+	-	+
As you get older	Cleft Palate Foundation	USA	+	-	-	+	-	+	+	+	+	+	+
Information for adults	Cleft Palate Foundation	USA	+	-	-	+	-	+	+	+	-	-	+
Feeding Your Baby	Cleft Palate Foundation	USA	+	-	-	+	+	-	-	-	-	-	+
Cleft surgery	Cleft Palate Foundation	USA	+	-	-	-	-	+	-	-	-	-	-
Help with Hearing	Cleft Palate Foundation	USA	+	-	-	+	-	+	-	+	+	-	-
Genetics and You	Cleft Palate Foundation	USA	+	-	+	+	-	-	-	-	-	-	-
Treatment options for better speech	Cleft Palate Foundation	USA	+	-	-	-	-	+	-	+	-	-	-
Parameters for evaluations and treatment of patients with CL/P or other craniofacial anomalies	Cleft Palate Foundation	USA	+	-	-	+	+	+	+	+	+	-	-
For Parents of Newborn Babies with Cleft Lip/Palate	Cleft Palate Foundation	USA	+	-	+	-	+	+	-	+	-	-	-
What about	Cleft Palate Foundation	USA	+	-	-	+	+	-	-	-	-	-	-

Breastfeeding?													
Choosing a Cleft Palate or Craniofacial Team	Cleft Palate Foundation	USA	+	-	-	+	-	-	-	-	-	-	-
Dealing with Your Insurance Company/HMO	Cleft Palate Foundation	USA	+	-	-	-	-	-	-	-	-	+	+
Financial Assistance	Cleft Palate Foundation	USA	+	-	-	-	-	-	-	-	-	+	+
Information about Submucous Cleft Palate	Cleft Palate Foundation	USA	+	-	-	+	+	+	-	+	-	-	-
Support For Individuals Affected by Cleft Lip and Palate	Cleft Palate Foundation	USA	+	+	-	+	-	-	+	+	+	-	+
